# The effectiveness of tele-rehabilitation on improvement of daily living activities in children with cerebral palsy: narrative review

**DOI:** 10.1186/s43161-021-00055-7

**Published:** 2021-12-24

**Authors:** Mohammad E. Tamboosi, Safeer S. Al-Khathami, Shamekh M. El-Shamy

**Affiliations:** 1 Department of Physical Therapy and Rehabilitation, King Abdulaziz Medical City, National Guard Hospital, Jeddah, Kingdom of Saudi Arabia; 2 Department of Physical Therapy, Security Forces Hospital, Makkah, Kingdom of Saudi Arabia; 3 Department of Physical Therapy, Faculty of Applied Medical Sciences, Umm Al-Qura University, Makkah, Kingdom of Saudi Arabia

**Keywords:** Tele-rehabilitation, Tele-health, Tele-medicine, Remote rehabilitation, Cerebral palsy, Children

## Abstract

**Aim:**

To investigate the effectiveness of tele-rehabilitation for children diagnosed with unilateral cerebral palsy.

**Method:**

The design of this study is a narrative review. An electronic search was conducted for studies that related to tele-rehabilitation using the following databases: CINAHL, PubMed, MEDLINE, OTSeeker, and PEDro. The data extracted were analyzed by evaluating them according to the key results, limitations, suitability of the methods used to the initial hypothesis, interpretation of the results, and impact of the conclusions in the field.

**Results:**

Out of 139 studies, 3 studies met the inclusion criteria. Further, manual searches of the references of included studies identified 2 more relevant studies. The interventions applied in those studies were web-based multi-modal therapy program using Move-it-to-improve-it (Mitii™), home-based hand-arm bimanual intensive therapy (H-HABIT), and lower-extremity functional training (LIFT). The outcomes were executive functions, occupational performance, activity capacity, dexterity, quality of bimanual hand-use, functional goals, gait capacity, and performance.

**Conclusion:**

Tele-rehabilitation is effective in improving the functions of the upper and lower extremities in daily living activities for children with unilateral cerebral palsy (UCP), aged between 2 to 18 years old, classified to levels I and II in GMFCS and levels I, II, and III in MACS. Webcam and good internet connection are essential requirements to conduct tele-rehabilitation. Children need to be contacted weekly via phone or e-mail for further follow-ups. Additionally, tele-rehabilitation may be considered one of the intervention strategies for patients who live in rural areas.

## Introduction

Cerebral palsy (CP) is a neurodevelopmental disorder that affects children’s development and limits their functions and activities. It occurs in 1.5 to 2.5 per 1000 live births [1]. CP is characterized according to the tone abnormalities and motor distributions abnormalities categorized as follows: [2] spastic CP (85–91%), [3] dyskinetic CP (4–7%), [4] ataxic CP (4–6%), and [5] hypotonic CP (2%). Dyskinetic, ataxic, and hypotonic CP mostly affect bilateral upper limbs and bilateral lower limbs, while spastic CP might be [2] hemiplegic CP which represent 38% of CP cases, diplegic CP (37%), and quadriplegic CP (24%) [6]. In Saudi Arabia, CP has the highest rate of prevalence among all children with neurological disorders (2.34 per 1000) [7].

The symptoms of CP include muscle tone abnormalities which are considered to be the most common symptoms among CP children. Hypertonicity, hyperreflexia, clonus, and poor coordination are also the symptoms of children diagnosed with CP. Other symptoms include feeding difficulties, drooling, hip dislocation, scoliosis, seizures, intellectual impairment, communication difficulties, and impaired vision and hearing; those symptoms cause many impairments for the child [8].

The most common impairment that CP children would have is upper limb impairment [9]. Another impairment that CP children would complain about is gross motor impairment. Those impairments reduce the speed of movement, accuracy, and coordination and cause a reduction in the independence of the activities of daily living (ADL) [10].

CP treatment depends on symptoms and impairments. The treatments have been divided into pharmacologic treatment (oral baclofen and botulinum toxin injection), which targets muscle tone abnormalities, and non-pharmacologic treatment, including surgical or rehabilitation treatment. Surgical treatment aims to reduce spasticity and improve gait kinematics, while rehabilitation programs may enhance motor development and help to prevent secondary deformities [11]. The outcomes of the surgical intervention may vary and could be difficult to be predicted [12].

Early intervention is essential for infants diagnosed with CP because infants who are not actively using their motor cortex may lose cortical connections and functions. An infant’s motor behavior increases by discovering and interacting with the environment. Hence, controlling and generating the growth, development of muscles, ligaments, and bones and development of the neuromotor system lead to improving the neuroplasticity [6]. Early interventions should include child-initiated movement combined with environment modification [13].

During pandemics and crises such as coronavirus (COVID-19), rehabilitation services would be almost impossible and difficult for children and their caregivers in the rehabilitation centers. One of the solutions that might be effective for children with disabilities for getting rehabilitation services is tele-rehabilitation [5].

Tele-rehabilitation has become an interest for many physical therapy professions especially during the crisis of COVID-19. World Confederation for Physical Therapy (WCPT) provided a global hub of resources to support the physiotherapy profession in its response to COVID-19, and advise the rapid, measured, and responsive way the global profession has responded, whereas tele-rehabilitation became a viable solution for offering rehabilitation services [5].

Tele-rehabilitation defines as a creative way to deliver rehabilitation services remotely using tele-communication technologies [14]. Communication between the therapist and the patient can be attained remotely by videoconferencing, e-mails, and texting [15]. Recent tele-communication technologies have given the chance for rehabilitation services to be delivered to the patients via the internet or other technology devices; research has shown effective improvement in a clinical outcome in cases with disabilities through tele-rehabilitation [2].

Recent studies have proven that tele-rehabilitation reinforces the early intervention for children diagnosed with CP [16]. Providing rehabilitation intervention at home (simulating the actual environment for the child) is better than having the intensive rehabilitation service at a day camp, which is the best motor skill that the child can learn [10].

Tele-rehabilitation might have many advantages such as reducing the cost for both therapists and patients and providing healthcare services for patients who live in rural areas and who have difficulties with transportation [15]. On the other hand, the required devices to provide therapeutic services are not flexible to be provided remotely [2]. Tele-rehabilitation could have some limitations because most of the interventions in the rehab centers included touching, handling, using machines such as treadmills, and performing specific tasks, which are difficult to be applied remotely [17]. Additionally, it is important to take the feedback from the patients and their caregivers to observe the outcomes during the rehabilitation that applied remotely [15].

Therefore, the purpose of this narrative review is to assess and synthesize the studies that used tele-rehabilitation to improve functions in daily activities. This will include investigating the effectiveness of tele-rehabilitation performed for children diagnosed with CP and investigate the psychometric properties of the outcome measures involved in the selected studies.

## Method

### Study design

The study design is a narrative review.

### Search strategy

An electronic search was conducted for studies that were published in English which is related to tele-rehabilitation using the following databases: CINAHL, PubMed, MEDLINE, OTSeeker, and PEDro from 2010 to September 2021, using the medical subject heading (MeSH) or keywords such as “tele-rehabilitation,” “tele-health,” “tele-medicine,” and “remote rehabilitation,” and every set search included MeSH terms which was combined by AND cerebral palsy. Table 1 summarizes the process of searching. A flow diagram was added to depict the flow of the information of the collected data.

**Table 1:** Keywords/terms used for electronic databases search

Database	Keywords
PubMed CINAHL MEDLINE	• Tele-rehabilitation AND cerebral palsy • Remote rehabilitation AND cerebral palsy • Tele-medicine AND cerebral palsy • Tele-health AND cerebral palsy
PEDro	• Telerehabilitation* cerebral palsy* • Telemedicine* cerebral palsy* • Telehealth* cerebral palsy*
OTSeeker	• Tele-rehabilitation • Tele-medicine • Tele-health

### Inclusion and exclusion criteria

The included studies were selected according to the following inclusion criteria: [2] children diagnosed with CP [3]; RCT designs, as they are given one of the highest levels of evidence in the hierarchies rank studies ([4] children aged between 2 and 18 years old [5]; studies that are published from 2010 to September 2021, to focus in recent technologies; and [18] studies measured the functional outcomes whether upper limbs functions or lower limbs functions.

On the other hand, the exclusion criteria were [2] children diagnosed with other impairments or diseases such as acquired brain injury, psychological problems, Erb’s palsy, autism, and Down’s syndrome [3]; interventions applied in rehabilitation centers or hospital settings [4]; the outcomes that measured were not reliable to assess functional status [5]; other studies rather than RCTs; and [18] studies published before 2010.

### Data extraction

The data extracted from each study were retrieved and include the citation details, participants’ characteristics, the interventions applied, the concluded outcomes, and the outcome measures that were performed to assess the improvement.

### Data analysis

The data were analyzed through the critical assessment process by evaluating them according to the following: [2] key results, [3] limitations, [4] suitability of the methods used to the initial hypothesis, [5] interpretation of the results, and [18] impact of the conclusions in the field, according to [19]. Additionally, the psychometric properties of the outcome measures were investigated.

## Results

### Literature search and screening process

**Fig. 1:**
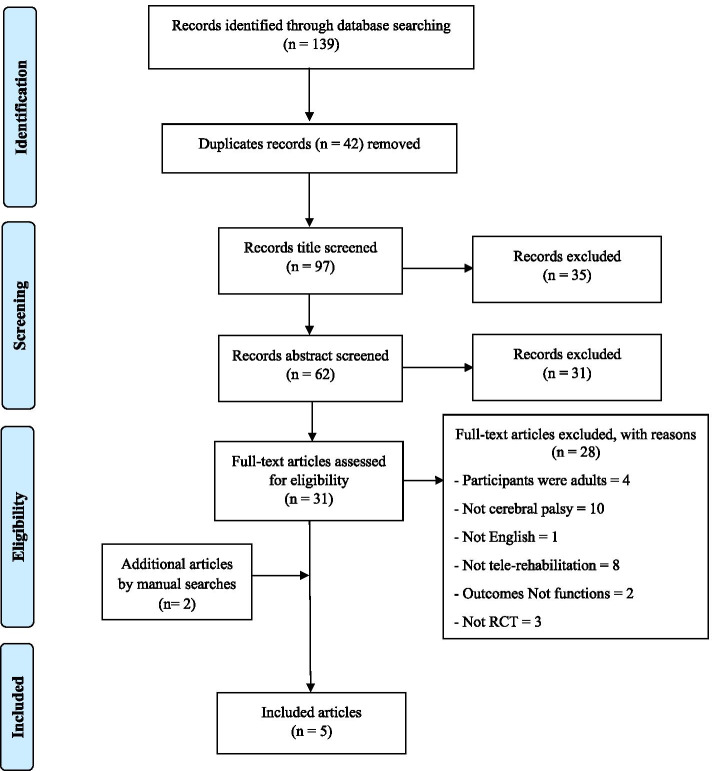
Flow diagram depicting search strategy and article selection process

The process of the search strategy and screening of articles that were followed in the current review is shown in Fig. 1. The electronic databases search retrieved 139 articles. After removing 42 duplicates, 97 articles were title screened which resulted in 62 studies, and these studies were abstract screened critically which resulted in 31 studies. Full-text screening of all relevant articles yielded 3 studies that satisfied the eligibility. Further, manual searches of the references of included articles identified 2 more relevant studies.

### Characteristics of the included studies

The main findings are summarized in Table 2. Tele-rehabilitation was performed in 353 children diagnosed with unilateral cerebral palsy (UCP); the range of the age was between 2 and 18 years old. They were classified to levels I and II according to the Gross Motor Function Classification System (GMFCS) and levels I, II, and III according to Manual Ability Classification System (MACS).

**Table 2:** Characteristics of the included studies

	Participant characteristics							
References	Number of participants	Age	Condition	MACS	GMFCS	Intervention	Outcomes	Outcome measure
James et al. [20]	N=101(52 males, 49 females)	8-17years	UCP	I, II & III	I, II	Mitii	activity capacity and performance	6MWT, ActiGraph GT3X+Mob- Ques28, LIFE-H
Mitchell et al. [21]	N=102matched in pairs	8-18years	UCP	I, II & III	I, II	Mitii	occupational performance	AMPS, AHA, JTTHF, MUULCOPM, TVPS-3,
M. Piovesana et al. [22]	N=102matched in pairs	8-18years	UCP	I, II & III	I, II	Mitii	Executive functions	WISC-IV-SF; D-KEFS; DSB & BRIEF
Ferre et al. [23]	N=24(14 females, 10 males)	2-13years	UCP	I, II & III	I, II	H. HABIT	Dexterity and bimanual hand function	BBT; AHA
Surana et al. [10]	N=24(14 females, 10 males)	2-13years	UCP	N/A	I, II	LIFT	Lower Extremity Function	1MWT, 10-Meter Walk Test, 30-s Chair Raise, single-leg stance & ABILOCO-kids

**Table 3:** Critical assessment process

References	Key results	Limitations	Suitability of the methods used to test the initial hypothesis.	Interpretation of the results.	Impact of the conclusions in the field.
James et al. [20]	The Mitiigroup demonstrated significantly greater post-intervention scores than the comparison groupon the AMPS, JTTHF dominant upper limb, COPM, and TVPS-3.	Few children reached 60 hours, the maximum target dose of. It may have that technical problems led to the lower-than-expected dose. The administrator of the JTTHF was not hidden to treatment allocation. Scoring of the AMPS was not blinded.	The hypothesis was that Mitii enhance the ADL and processing skills to achieve occupational performance goals and visual perceptual skills. Mitii demonstrated a significant improvement in ADL and processing skills. Thus, the occupational performance goals and visual processing were improved.	Web-based therapy can be performed at home and has the ability to improve the dose of therapy. Mitii can be an option for children with UCP to improve their occupational performance and visual perception.	Mitii can improve the ADL, occupational performance, and visual perception. However, it will not improve the upper extremities functions.
Mitchell et al. [21]	A significant improvement in functional strength and 6MWT distance	There was no significant improvement in the activity efficiency or entertainment participation, it did not decrease the mobility limitations as well.	Those who obtained training would have higher activity ability and efficiency. The functional strength and walking Endurance were increased	Training showed a good result in increasing functional ability and walking endurance in ambulant children. nonetheless, there was no efficacy of the general activity	The use of a web-based program to independently ambulant children with UCP was successful in increasing activity potential, as measured by functional strength and walking endurance.
M. Piovesana et al. [22]	There were no significant differences in attentional control, cognitive flexibility, problem solving, information processing or executive function performance	There were few participants who reached the proposed target dose of 60 hours, with the average of 32.4h. They had a technical issue regarding the intervention in preventing some participants to reach the maximum dose. The continues issues regarding the internet connection resulted a difficulty to access the program which lead to stop the sessions in some cases and redo the session in other time.	They hypothesized that Mitii improves the capacity of executive function. But the hypotheses were not supported.	Mitii demonstrated no significant improvement in executive functions. In contrast, it showed an improvement in the motor skills and processing skills, activities of ADL, and physical capacity, which could be an effective web based multimodal therapy for these functions.	Using Mitii to treat executive functions is not feasible, but it is effective in improving the motor skills and processing skills, activities of ADL, and physical capacity.
Ferre et al. [23]	H-HABIT showed greater improvement on BBT but no improvement on AHA compared with control group.	strong attrition was recorded (the 6 months follow up was not completed by 3 children from the control group). There were no clear instructions regarding a set time for the training during the day which might affect the result of the study.	The hypothesis was that H-HABIT improves the dexterity, bimanual hand-use effectiveness and functional goals compared to control group, the hypothesis was confirmed in the results.	H-HABIT improves children’s dexterity and performance, but does not improve the bimanual performance, compared to a control group.	Home-based programs provide a valuable, family-centered approach for increasing the intensity of the rehabilitation. Performing H-HABIT at home improves dexterity and upper extremities functions
Surana et al. [10]	LIFT group demonstrated a significant improvement in 1MWT, and ABILOCO-kids compared with the control group	They faced high attrition (the program was not completed by 8 participants and 6 were dropped out). The activities outside the training hours were not controlled, which prevent the disruption of the psychosocial dynamics. The gait capacity was not measured.	They hypothesized that LIFT would demonstrate a greater improvement in gait and LE functions compared with control group, but the gait was not measured.	They mentioned that LIFT group showed increased distances in gait which led to improvement in gait capacity. The ability to move in the surrounding environment was assessed using ABILOCO-kids, the results showed an improvement in gait capability and performance.	performing LIFT at home showed its effectiveness to improve gait capacity and performance.

The interventions were applied remotely including a web-based multi-model therapy program (Mitii), home-based hand-arm bimanual hand function intensive therapy (H-HABIT), and lower-extremity intensive functional training (LIFT). The main outcomes were functional outcomes including executive function, occupational performance, activity capacity, dexterity, bimanual hand function, and lower extremity function. The selected studies were assessed critically using the critical assessment process; Table 3 demonstrates the critical assessment in detail.

### Web-based multi-modal therapy program

The studies ([20, 21], and [22] used Move-it-to-improve-it (Mitii), a multi-modal web-based program as a tele-rehabilitation to determine the effectiveness of the program to improve activity capacity, occupational performance, and executive function respectively in children with UCP compared with the control group (waitlist).

All the previous studies applied for the program in the home setting, and they were monitored via a computer and webcam. The duration of the intervention was performed for 20 weeks, 20–30 min per day, 6 days per week. They were contacted weekly via phone or e-mail to provide feedback and support. The outcome measures were taken remotely for both groups, at the baseline and after 20 weeks.

The program was varied according to the child’s needs. However, it included 40% of gross motor exercises such as squatting on balance foam by pretending to fly on a spaceship or shooting a pirate ship with a cannonball to perform for lunging. On the other hand, roughly 75% of the lower limb strengthening exercise was determined by task setting. The intensity of the exercise was increased weekly by increasing the repetition and speed of the task and height of the step.

Mitii showed no significant improvement in executive functions for children with UCP. In contrast, it showed an improvement in the motor skills and processing skills, activities of daily living, occupational performance, processing skills, goal attainment, visual processing, and physical capacity in children with UCP.

### Home-based hand-arm bimanual intensive therapy (H-HABIT)

The study [23] used H-HABIT to investigate the efficacy of H-HABIT to improve the dexterity, functional performance, and bimanual performance in children with UCP compared with lower-limb intensive functional training (LIFT-control). Participants were monitored remotely via webcam software (Adobe Connect) during performing the exercises at home.

Children performed home-based activities under caregivers’ supervision for 2 h per day, 5 days per week, for 9 weeks. The H-HABIT group performed exercises in form of children’s fun sports including bimanual specific activities, environmental shaping to enhance bimanual hand-use, and grading of desired tasks. In LIFT-control, the exercises were performed as a fun game during functional tasks including ball kicking, hopscotch game, or walking over obstacles.

They hypothesized that H-HABIT would improve dexterity, quality of bimanual hand-use, and functional goals; the study interpreted that the H-HABIT program improves the dexterity and performance of functional goals, but there was no improvement in bimanual performance. Additionally, home-based rehabilitation programs provide a valuable family-centered approach to increase the rehabilitation intensity.

### Lower-extremity intensive functional training (LIFT)

In the study [10], the effectiveness of LIFT was investigated compared with the control group which received H-HABIT to improve the gait and gross motor functions by improving the strength of the lower extremity and enhance the balance and coordination. The activities were monitored remotely using Adobe Connect once a week for 1 h.

LIFT was used to determine if there were any improvements in motor skills or strength. Strengthening domain included body weight exercises such as sit to stand, step up, and jumping; regarding the gait (motor skills), the lower extremities muscle group was targeted to be strengthened using body weight and thera-band such as bridging and clamshells. Regarding balance, children performed activities such as single-leg standing, and standing on unsteady surfaces, ball kicking, tandem walking, jumping jacks, and galloping/skipping to improve coordination. The program was performed daily, 2 h/day.

They concluded that intensive intervention for lower extremity applied at home environment given by caregivers has a significant improvement in gait capacity and performance. Tele-rehabilitation program using LIFT with remote supervision resulted in an improvement in ambulation distance and overall walking ability.

### Outcome measures

The outcome measures used in the included studies were briefly described and investigated according to the population (condition and age) and psychometric properties (validity and reliability). Table 4 demonstrates the details regarding the results of the outcome measure investigation.

**Table 4:** Outcome measures description and psychometrics

Measurement tool	Description	Population		Psychometric properties		Reference
		Condition	Age	Validity	Reliability	
Wechsler Intelligence Scale for Children, Fourth Edition Short-Form (WISC-IV-SF)	This tool is a cognitive assessment tool to measure the four index scores and Full-Scale IQ	Children with intellectual impairment	6 -16 years	High construct validity	Excellent reliabilityICC= 0.97	Crawford et al. [24]
Delis–Kaplan Executive Function System (D-KEFS)	Designed to Assess the integrity of the frontal system of the brain.Determine how deficits in abstract, creative thinking may impact upon an individual’s daily life.D-KEFS offers two forms: Standard Record Forms include all nine D-KEFS tests, while the Alternate Record Forms include alternate versions of D-KEFS Sorting, Verbal Fluency, and 20 Questions Tests.	Mild cognitive impairmentsubcortical ischemic diseaselateralized right-hemisphere damageParkinson’s diseasesmultiple sclerosisautismAsperger’s syndrome	Children and adults from 8 - 89 years	More research is needed for this test regarding its sensitivity to deficits in verbal abstraction skills	Good reliabilityICC=0.80	Delis et al. [25]
Behavior Rating Inventory of Executive Function (BRIEF)	Ecological measure forthe assessment of executive functions.The questionnaire ismade up of 63 items on five clinical scales.It takes approximately 10 to 15 min to complete.	Children identified with ADHD	2 - 18 years	Moderate convergent validity with psychopathology and temperament, which considered valid with ADHD	Good reliability(ICC = 0.86-0.95)	Ezpeleta et al. [26]
Box and Blocks Test (BBT)	Assess gross manual dexterityIt is a quick, simple, and inexpensive test.It can be used with a wide range of populations including Neuromuscular Disorders.	Children with upper limb impairment	6- 19 years	Construct validity	Excellent test–retestreliability, inter/intrarater reliabilityICC= 0.97	Araneda et al. [3]
Assisting Hand Assessment (AHA)	Describes how effectively children with unilateral disability, in particular children with congenital unilateral disability.	Children with UCPor obstetric brachial plexus palsy	1yr and 6mth - 12 years	Construct validity	Excellent reliabilityICC= 0.99	Holmefur & Krumlinde-Sundholm [27]
1-minute walk test (1MWT)	Potential measure of functional ability and walking endurance.	Children with ambulatory CP	4 - 16 years	Concurrent validity	Excellent test-retest reliabilityICC=0.94	McDowell et al. [28], Martakis et al. [29]
10-Meter Walk Test	Measure locomotor capacity in clinical and research settings.	Children and adolescents with neurological dysfunction	2 - 12 years	High validity in the neurological diseases	Good reliability0:70 < ICC > 0:89	de Baptista et al. [30]
30-s chair rise	The 30-Second Chair Test is administered using a chair without arms, with height of 43.2 cm. The chair, with rubber tips on the legs, is placed against a wall to prevent it from moving.	People with osteoarthritis	18 - 64 years	Concurrent criterion validity	Good test-retest reliabilityICC=0.87	Silva et al. [31]
single-leg stance (SLS)	(SLS) Test is used to assess static postural and balance control.These protocols usually include eyes open and eyes closed test-variations	Children with musculoskeletal birth defects such as Congenital talipes equinovarus	4 -18 years	Construct Validity	Excellent reliabilityICC= 0.81- 0.91	Sember et al. [32]
ABILOCO-kids	Assessing the walking ability of children with CP focusing on the activity domain of the ICF	Children with CP	6 - 15 years	Construct validity	Excellent reliabilityICC= 0.96	Gilles et al. [33]
6-minute walk test (6MWT)	Used to define their capacities and to evaluate therapeutic interventions.	Children with CP	4 - 18 years	Criterion Validity	Excellent test-retestICC= 0.98	Fitzgerald et al. [34]
28-item Mobility Questionnaire (Mob- Ques28)	scores mobility limitations on a scale of 0 to 100	Children with CP	2 - 13 years	Good validity	Good reliabilityICC=0.87	Van Ravesteyn et al. [35]
ActiGraph GT3X+	The initialization is to record the number of steps and acceleration at a frequency of 100 Hz within 5 seconds.	Children with CP	6 - 14 years	Concurrent validity	Excellent reliabilityICC= 0.96-0.99	O’Neil et al. [36]
Assessment of Life Habits (LIFE-H)	Evaluate common task skills and obtain a domain-weighted score of 0 to 10.	Children with disabilities	5 - 13 years	Concurrent validity	Good reliabilityICC=0.80	Noreau et al. [37]
Assessment of Motor and Process Skills (AMPS)	Rasch-analyzed, observational evaluation of ADL motor and processing (not free). The AMPS is not suitable for children under the age of 3	Children with a wide range of diagnoses	3 - 15 years	Construct validity	Excellent test-retest and interrater reliabilityICC=0.90	Payne & Howell [38]
Jebsen–Taylor Test of Hand Function (JTTHF)	Evaluated upper limb unimanualspeed and dexterity.	Children with CP	5 - 17 years	Construct validity	Good to excellent test retest reliabilityICC= 0.84-0.97	Sığırtmaç & Öksüz [39], Araneda et al. [3]
Melbourne Assessment of Unilateral Upper Limb Function (MUUL)	Measure the quality of the impaired upper limb by its reach, grasp, release, and manipulation.	Children with neurological impairment.	5 - 16 years	Criterion validity	Excellent reliabilityICC=0.96	Randall et al. [40]
Canadian Occupational Performance Measure (COPM)	Evaluates self-perceived occupational performance in five areas identified by child and caregivers.	Children with disabilities	1 - 7.5 years	Construct validity	Good reliabilityICC=0.76-0.89	Verkerk et al. [41]
Test of Visual Perceptual Skill (non-motor) 3rd edition (TVPS-3)	Evaluates visual perception across 7 domains (visual discrimination, spatial relations, visual memory, form constancy, sequential memory, figure-ground discrimination, and visual closure).	Children with visual-perceptual problems	4 - 12 years	Construct validity	Good reliabilityICC=0.88	Chan & Chow [42]

## Discussion

This narrative review aims to evaluate and synthesize studies that used tele-rehabilitation to improve functions in daily activities for children with CP. This involved investigating the effectiveness of tele-rehabilitation for children with CP.

During pandemics and crises, tele-rehabilitation is the best solution that seems to be an effective treatment for children with disabilities during the pandemic; World Confederation for Physical Therapy (WCPT) provided a global hub of resources to support the physiotherapy profession to continue the rehabilitation programs during the pandemic [5]. The study [16] demonstrated that tele-rehabilitation is an alternative intervention to the usual rehabilitation at the rehabilitation center for children who live in rural areas and have transportation difficulties. Nevertheless, the frequency and duration of the rehabilitation intervention impact the improvement of the outcomes; the proper contact via phone or e-mail with the caregivers, at least once a week, may lead to greater improvements [18].

The effectiveness of tele-rehabilitation might have the same outcomes as compared to interventions that are applied face-to-face [18]. However, to promote effective tele-rehabilitation for children with CP, good internet connection is required to avoid the technical issues that occurred in the included studies ([20–22]. Furthermore, the included studies in our review did not mention any risk of injury that may be associated with tele-rehabilitation, as the caregivers have no experience regarding the proper rehabilitation for children diagnosed with CP.

This narrative review showed that tele-rehabilitation has significant effectiveness for children with UCP to improve functions (gait capacity and performance, occupational performance, dexterity, bimanual hand function, and lower extremity function); the age range was between 2 and 18 years old, levels I and II in GMFCS, and levels I, II and III in MACS. However, children with younger age were not included. Also, children with levels III, IV, and V in GMFCS and levels IV and V in MACS were not included as there is no evidence to support this hypothesis, which indicates certain barriers to be encountered in such cases. We have also noticed that all the selected studies included children diagnosed with UCP, which reflects that the result of our study can be applied to children with UCP only and not for all children with CP. In contrast, tele-rehabilitation has a significant improvement for other cases in other studies that were not included in our study as we focused only on CP cases.

From our point of view, treating children with levels III, IV, and V in GMFCS and levels IV and V in MACS might need special equipment and special techniques that the caregivers are unable to perform them as they do not have enough experience, which corresponds to the study [17], that tele-rehabilitation could have some limitations because most of the interventions in the rehab centers included touching, handling, or using machines and equipment such as treadmills or special tools to perform specific exercises, which are difficult to be applied remotely.

During investigating the outcome measures, we found that some of the included studies used invalid measurement tools for children aged from 2 to 18 years old. For example, BBT is a tool designed to assess gross manual dexterity for children aged between 6 and 19 years old [3]. Thus, the measurement tool is invalid for children younger than 6 years. Also, the 30-s chair rise is a test performed for adults with osteoarthritis [31] indicating that the outcome measure used in that study is invalid for young children. These outcome measures lead us to re-consider about the outcome measures that are supposed to be used in the field.

Given the above limitations, we are unable to generalize that tele-rehabilitation is effective for all children with CP just because the selected studies specified children with levels I and II GMFCS and levels I, II, and III on MACS.

### Limitations of the study

This study is a narrative review; a risk of bias or quality assessment of the included studies was not performed in this review. Additionally, the included studies were all RCTs because they are given the highest level of evidence in the hierarchies rank studies. Furthermore, there were clear methodological limitations in the included articles which could have influenced the findings of this review, for example, the selected studies did not include children with moderate or severe disability given that all children were levels I and II on GMFCS and levels I, II, and III on MACS, which affect on the results of the treatment for children with moderate or severe disabilities. Also, the included studies did not mention any risk of injury that may be associated with tele-rehabilitation, which may influence the treatment and cause complications and difficulties for the patients.

### Recommendations for research

Further studies are recommended by performing a quality assessment for the selected studies in form of a systematic review. Additionally, it is recommended to include other studies rather than RCTs for further investigation regarding the effectiveness of tele-rehabilitation. Risk of injury associated with tele-rehabilitation should also be investigated. All levels of disability children are suggested being included.

## Conclusion

The findings of this narrative review showed that tele-rehabilitation is effective in improving the functions of the upper and lower extremities in daily living activities (gait capacity and performance, occupational performance, dexterity, bimanual hand function, and lower extremity function) for children diagnosed with UCP, aged between 2 and 18 years old, classified to levels I and II in GMFCS and levels I, II, and III in MACS.

Children should be monitored via a webcam and good internet connection. In addition, they should be supervised by therapists for at least once weekly via a phone, e-mail, or video conference to promote an effective tele-rehabilitation. The suggested duration and intensity are 30 min for 5 days weekly regarding the Mitii and 2 h for 5–6 days regarding performing LIFT or H-HABIT. Additionally, tele-rehabilitation may be considered one of the intervention strategies for patients who live in rural areas. Tele-rehabilitation should be performed with caution; the children should be monitored and followed up frequently to prevent the risks of injury that may occur during performing the suggested intervention that is performed remotely.

## Data Availability

No data and materials are available for this review.

## Abbreviations

1MWT: 1-Minute Walk Test
30-s chair rise: 30 Seconds Chair Rise
6MWT: 6-Minute Walk Test
ABILOCO-Kids: A Measure of Locomotion Ability for Children
ADL: Activities of daily living
AHA: Assisting Hand Assessment
AMPS: Assessment of Motor and Process Skills
BBT: Box and Blocks Test
BRIEF: Behavior Rating Inventory of Executive Function
COPM: Canadian Occupational Performance Measure
COVID-19: Coronavirus disease 2019
CP: Cerebral palsy
D-KEFS: Delis–Kaplan Executive Function System
GMFCS: Gross Motor Function Classification System
H-HABIT: Home-Based Hand-Arm Bimanual Intensive Therapy
JTTHF: Jebsen–Taylor Test of Hand Function
LIFE-H: Assessment of Life Habits
LIFT: Lower-Extremity Functional Training
MACS: Manual Ability Classification System
Mitii: Move-it-to-improve-it
Mob- Ques28: 28-item Mobility Questionnaire
MUUL: Melbourne Assessment of Unilateral Upper Limb Function
N: Number
N/A: Not applicable
SLS: Single-leg stance
TVPS-3: Test of Visual Perceptual Skill (non-motor) 3rd edition
UCP: Unilateral cerebral palsy
WISC-IV-SF: Wechsler Intelligence Scale for Children, Fourth Edition Short-Form
